# Genetic association of NOS1 exon18, NOS1 exon29, ABCB1 1236C/T, and ABCB1 3435C/T polymorphisms with the risk of Parkinson's disease: A meta-analysis

**DOI:** 10.1097/MD.0000000000004982

**Published:** 2016-10-07

**Authors:** Hongbin Huang, Cong Peng, Yong Liu, Xu Liu, Qicong Chen, Zunnan Huang

**Affiliations:** a Key Laboratory for Medical Molecular Diagnostics of Guangdong Province, Dongguan Scientific Research Center, Dongguan, Guangdong; b The Second School of Clinical Medicine, Guangdong Medical University, Dongguan, Guangdong; c Hunan key Laboratory of Skin Cancer and Psoriasis, The Department of Dermatology, Xiangya Hospital, Central South University, Changsha, Hunan; d School of Pharmacy, Guangdong Medical University, Dongguan, Guangdong; e Key Laboratory for Research and Development of Natural Drugs of Guangdong Province, Zhanjiang, Guangdong; f Department of Biochemistry and Molecular Biology, Guangxi Medical University, Nanning, Guangxi, PR China.

**Keywords:** ABCB1, meta-analysis, NOS1, Parkinson's disease, polymorphism, statistically significant correction

## Abstract

**Background:**

Parkinson's disease (PD) is the second most frequent neurodegenerative disorder. Previous publications have investigated the association of NOS1 and ABCB1 polymorphisms with PD risk. However, those studies have provided some contradictory results.

**Methods:**

Literature searches were performed using PubMed, Embase, PDgene, China National Knowledge Infrastructure database, and Google Scholar. Odds ratios (ORs) with 95% confidence intervals (CIs) were applied to evaluate the strength of association.

**Results:**

The analysis results indicated that NOS1 exon18 polymorphism was associated with developing PD in 4 genetic models (allelic: OR = 1.25, 95%CI 1.09–1.44, *P* = 0.001; homozygous: OR = 1.79, 95%CI 1.32–2.45, *P* < 0.001; recessive: OR = 1.70, 95%CI 1.26–2.28, *P* < 0.001; dominant: OR = 1.22, 95%CI 1.02–1.46, *P* = 0.03), whereas exon29 polymorphism was not correlated to PD susceptibility. In addition, ABCB1 1236C/T polymorphism was related to PD in the recessive (OR = 0.80, 95%CI 0.66–0.97, *P* = 0.025) and overdominant (OR = 1.21, 95%CI 1.03–1.43, *P* = 0.02) models, which might indicate the opposite effects of 2 minor variants of this locus on Parkinson's disease. However, this associated result was not robust enough to withstand statistically significant correction. On the other hand, no association was found between ABCB1 3435C/T polymorphism and the predisposition to PD in 5 genetic models, and such an absence of relationship was further confirmed by subgroup analysis in Caucasians and Asians. Whether the polymorphisms of these 4 loci were linked to PD or not, our study provided some interesting findings that differ from the previous results with regard to their genetic susceptibility.

**Conclusion:**

The NOS1 exon18 and ABCB1 1236C/T variants might play a role in the risk of Parkinson's disease, whereas NOS1 exon29 and ABCB1 3435C/T polymorphisms might not contribute to PD susceptibility.

## 1 Introduction

Parkinson's disease (PD), regarded as a common incurable neurodegenerative disease, influences around 1% of the worldwide population above age 60.^[1–3]^ Patients suffering from PD have faced many problems in their daily life, such as a life of low quality, an economic burden of health care, and a collapse of physical and emotional well-being. Besides, the increasing numbers of PD patients have a negative effect on the development of society and economy.^[4,5]^ The pathological characteristics of PD include the loss of dopaminergic neurons in the substantia nigra pars compacta and the buildup of α-synuclein in Lewy bodies.^[6,7]^

The pathogenesis of Parkinson's disease could be attributable to genetic, environmental, or other factors.^[8]^ The genetic background of Parkinson's disease was well established,^[9]^ in which the monogenic forms could influence the development of Parkinson's disease. Recently, the polymorphisms of several genes, such as CYP1A1, CYP1A2, ABCB1, PON1, PON2, and NOS1,^[3,10]^ were considered as the candidate risk factors for Parkinson's disease. In this study, we focused on the NOS1 and ABCB1 gene polymorphisms with the risk of PD.

The NOS1 gene is located at chr12q24.2 to chr12q24.3, which is the first isoform found in neurons. The nNOS (NOS1) is calcium (Ca^2+^)-dependent and its isoforms are constitutively expressed in many tissues, which include vessels and neurons.^[11,12]^ In addition, the NOS1 can control a variable low level of nitric oxide (NO) to carry out normal physiological functions in the neurons.^[13]^ NO is also a pro-oxidant capable of adding oxidative/nitrosative stress that can damage neurons. Therefore, it is possible that Parkinson's disease is susceptive to the polymorphisms of NOS1.

The ABCB1 gene, also recognized as MDR1, is located on chromosome 7q21.1. The ABCB1 gene is widely expressed in human organs and tissues, such as capillaries of the brain.^[14–16]^ It encodes the P-glycoprotein (P-gp), a transmembrane protein, which regulates the brain entry of various xenobiotic. The P-gp belongs to a highly preserved superfamily of ATP-binding cassette (ABC) transporters. This protein is present at the blood–brain barrier where it functions as a drug transporter.^[17–19]^ ABCB1 acts as an efflux transporter for many substrates such as chemotherapeutic agents, anti-epilepsy medicine, or drug and antibiotics for PD.^[20–22]^ Thus, the function disorder of ABCB1 gene may be a risk factor for Parkinson's disease.

Previous publications have explored the connection of 2 single nucleotide polymorphisms (SNPs) of NOS1^[23]^ and several SNPs of ABCB1^[18,24–33]^ with PD. Here, we studied the effect of the 4 genetic polymorphisms, exon18 and exon29, in NOS1, and rs1128503 (1236C/T), rs1045642 (3435C/T) in ABCB1 on the predisposition to Parkinson’ disease. We did not investigate other polymorphisms in ABCB1, such as rs1202169 and rs2235035, because those polymophisms lacked enough case-control studies for meta-analysis.^[18,27,34,35]^

To date, no meta-analysis has been carried out to estimate the association of NOS1 exon18, exon29, and ABCB1 1236C/T polymorphisms with the susceptibility to PD. Although a previous meta-analysis explored the connection between ABCB1 3435C/T and PD risk, only 2 articles were included in that study, which might lack the statistical power to identify the true relationship. Besides, the original results of the previous case-control studies^[18,24–33]^ were inconsistent. Hence, we conducted this meta-analysis based on all currently available case-control studies to further examine whether these 4 polymorphisms were potentially associated with the risk of Parkinson’ disease.

## 2 Materials and methods

### 2.1 Search strategy

Literatures were searched from PubMed, Embase, and China National Knowledge Infrastructure (CNKI). With the purpose of getting as many potentially relevant publications, we used the following keywords including “(NOS or NOS1 or nitric oxide synthase 1 neuronal) AND (Parkinson's disease or Parkinsonism) AND (polymorphism or mutation or variation or variant)” AND “(ABCB1 or MDR1) AND (Parkinson's disease or Parkinsonism) AND (polymorphism or mutation or variation or variant)”. In addition, we explored the PD Gene database (http://archive.pdgene.org/default.asp) as well as Google Scholar, and also conducted a manual search of references in the individual articles to avoid the missing of some related publications. All relevant publications were scanned on the basis of title, keywords, and abstract, and the irrelevant ones were excluded after the full text of the articles was further read. The literature search was updated on April 28, 2016.

### 2.2 Inclusion and exclusion criteria

Studies were included if they met the following criteria: (1) in a case-control design, (2) on the association between 4 polymorphisms (exon18, exon29, 1236C/T, and 3435C/T) and the risk of PD, (3) with complete genotype data. The exclusion criteria were as follows: (1) duplicate research, animal studies, and review articles, (2) no case-controls studies, case-only studies, or control-only studies, (3) studies for other diseases, genes, and polymorphisms, (4) studies without sufficient genotype data. Two reviewers extracted eligible studies independently and any disagreement of the included articles was resolved by discussion among the authors.

### 2.3 Data extraction

From the retrieved studies, we extracted the following information: author's name, publication year, study area, participant ethnicity, the number of PD cases and controls, the number of genotypes of NOS1 (exon18 and exon29) and ABCB1 (1236C/T and 3435C/T) polymorphisms, Hardy–Weinberg equilibrium (HWE), and source of included articles.

### 2.4 Quality assessment

The Newcastle–Ottawa Scale criteria^[36]^ were applied to evaluate the quality of eligible studies on the basis of 3 aspects: selection, comparability, and exposure. NOS scores ranged from 0 to 9, which being no less than 6 indicated high quality.

### 2.5 Statistical analysis

STATA statistical software (Stata 14.0) and Review manager (version 5.2) were used to evaluate the available data from each study. The strength of association between any of NOS1 exon18, NOS1 exon29, ABCB1 1236C/T, and ABCB1 3435C/T polymorphisms was assessed by combined odds ratio (OR) and 95% confidence interval (CI).^[37]^ The significance of OR was determined with the Z-test, and *P* < 0.05 was regarded as statistically significant. The reported *P* was adjusted by Bonferroni–Holm correction (BON)^[38]^ and Benjamini–Hochberg False Discovery Rate (FDR)^[39]^ methods to control the false discovery rate. The degree of heterogeneity between studies was evaluated by the *Q*-test and *I*^*2*^-statistics. The fixed-effect model was used if *P* > 0.05 or *I*^*2*^ < 50%. Otherwise, the random-effect model was utilized.^[40]^ Subgroup analysis was used to explore the reasons for heterogeneity. Publication bias was investigated by Begg's and Egger's test and the potential bias was found by *P* < 0.05.^[41,42]^ Sensitivity analysis was performed by excluding individual studies in sequence to assess the stability of the meta-analysis results. The quality of genotype data was estimated by Hardy–Weinberg equilibrium (HWE) and low-quality studies deviated from HWE were excluded in the sensitivity analysis.

### 2.6 Ethics statement

Ethical approval was not required for this study, as it is a systematic review and meta-analysis. This work was conducted according to the PRISMA (Preferred Reporting Items for Systematic Reviews and Meta-Analyses) guidelines.^[43]^

## 3 Results

### 3.1 Detection and selection of studies

**Figure 1:**
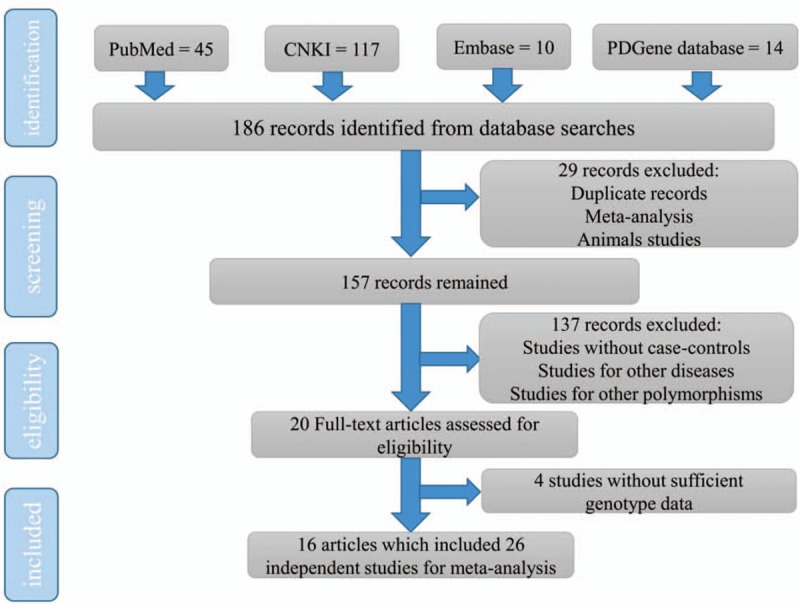
A diagram to describe the selection procedure of the eligible studies.

The process of the literature search and selection was discussed in Fig. 1. Initially, our search strategy yielded 186 possibly relevant papers. After removing 29 studies for duplications, animal studies, and meta-analysis, 157 studies remained. Among them, a total of 137 studies did not meet the inclusion criteria, such as studies not in a case-control design or related to other diseases or polymorphisms. They were excluded and we obtained 20 full-text articles. After that, 4 studies were further deleted due to their insufficient genotype data. Finally, we got 16 articles which included 26 independent studies in our meta-analysis. Among these 26 individual studies, 4, 5, 5, and 12 studies were linked to NOS1 exon18, NOS1 exon29, ABCB1 1236C/T, and ABCB1 3435C/T polymorphism, respectively. The basic characteristics of all the eligible articles were reviewed in Table 1. All these included studies conformed to HWE. In addition, the NOS result shows the score of each study reached 6 points or more (Table 2). Therefore, all these studies in our meta-analysis were high quality.

**Table 1:**
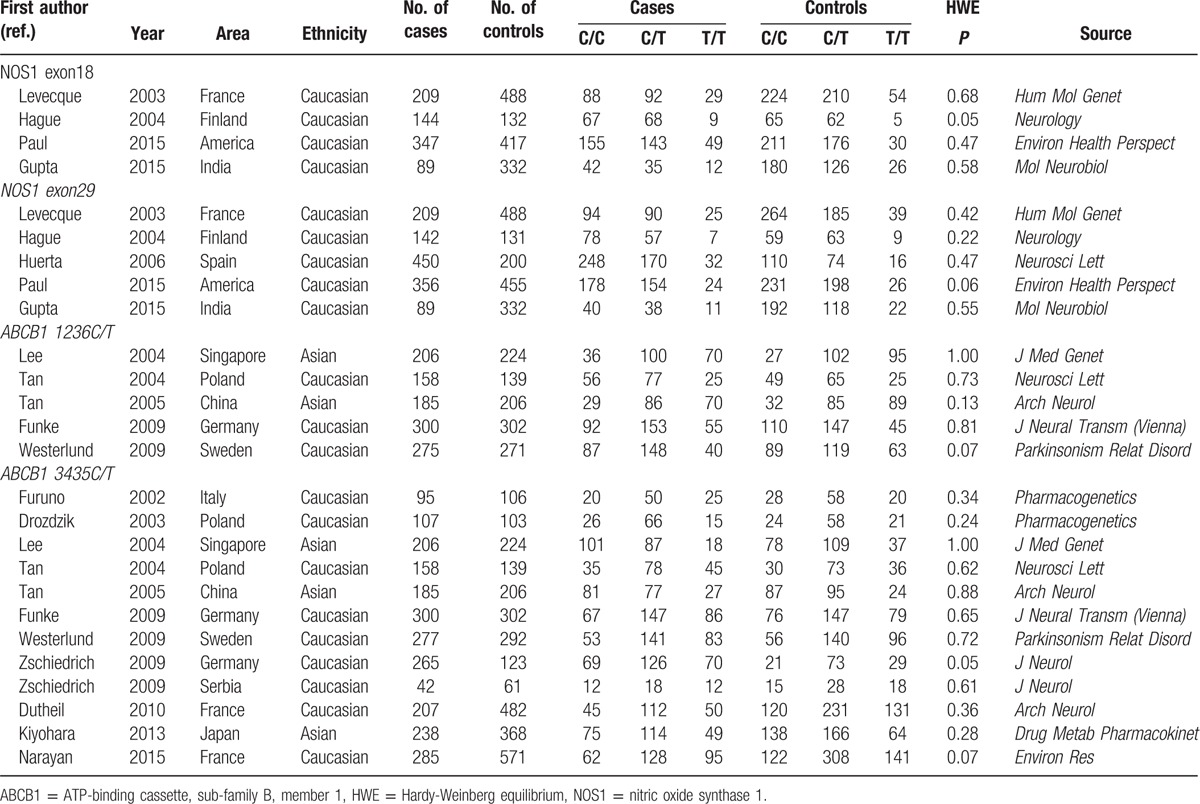
The basic characteristics of all included articles.

**Table 2:**
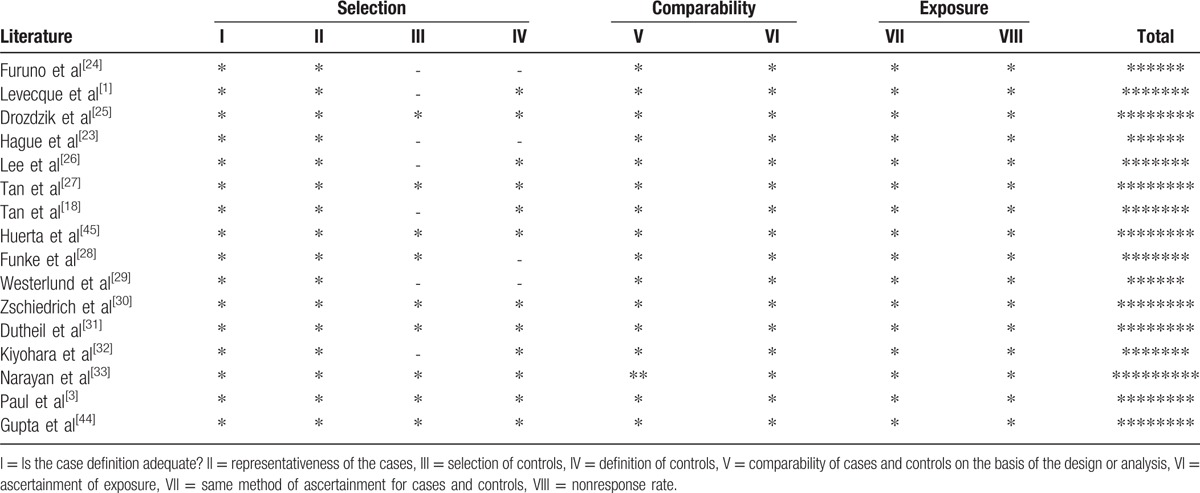
Quality assessment scheme for included literatures (Newcastle–Ottawa Scale).

### 3.2 Association of NOS1 exon18 polymorphism with the risk of Parkinson's disease

In this meta-analysis, we enrolled 4 articles^[1,3,23,44]^ including 789 cases and 1369 controls to investigate the role of NOS1 exon18 polymorphism in Parkinson's disease. The result was shown in Table 3. Although no connection was found in the heterozygous model (TC vs CC: OR = 1.11, 95%CI: 0.92–1.35, *P* = 0.27), NOS1 exon18 polymorphism was observed to be statistically significantly associated with the development of PD in other 4 genetic models (allelic T vs C: OR = 1.25, 95%CI 1.09–1.44, *P* = 0.001; homozygous TT vs CC: OR = 1.79, 95%CI 1.32–2.45, *P* < 0.001; recessive TT vs TC + CC: OR = 1.70, 95%CI 1.26–2.28, *P* < 0.001; dominant TT + TC vs CC: OR = 1.22, 95%CI 1.02–1.46, *P* = 0.03) (Table 3). The association was still significant according to the adjusted *P* calculated from the Bonferroni–Holm correction and FDR methods (also as shown in Table 3). The analysis indicated that NOS1 exon18 polymorphism was a risk factor for PD.

**Table 3:**
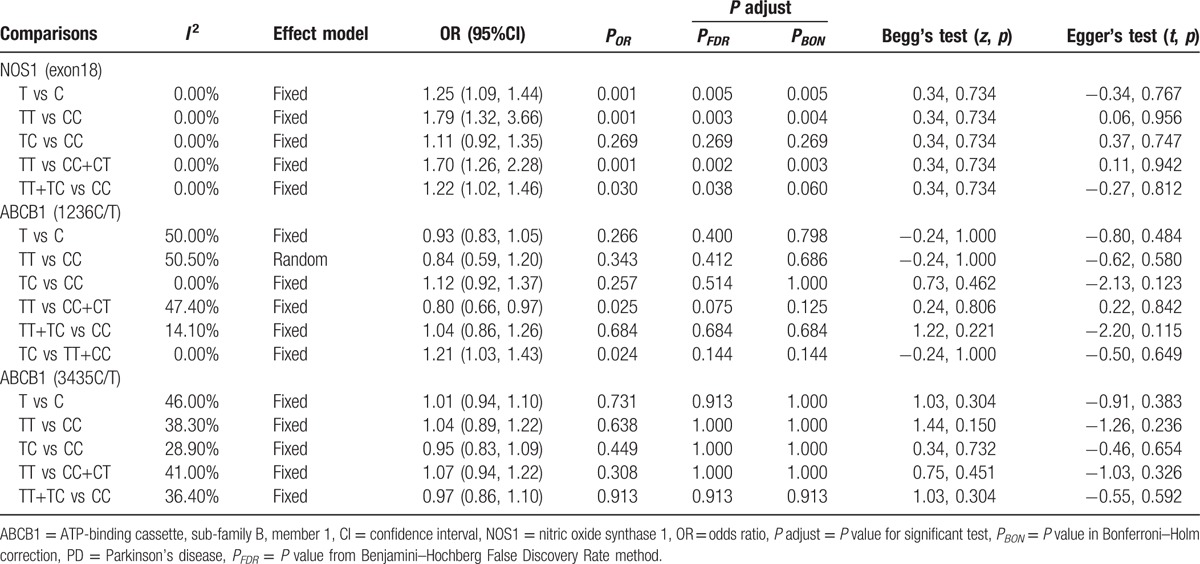
Meta-analysis of NOS1 exon18, ABCB1 1236C/T, and ABCB1 3435C/T with the PD risk.

### 3.3 No association between NOS1 exon29 polymorphism and the susceptibility to Parkinson's disease

**Figure 2:**
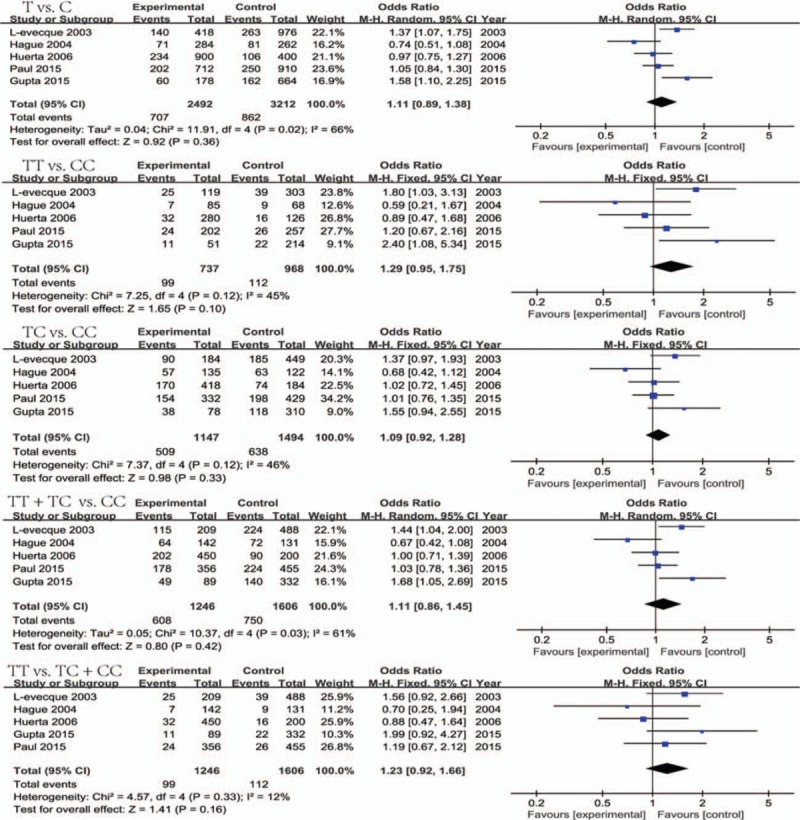
Forest plots of NOS1 exon29 polymorphism and PD risk in 5 genetic models. NOS1 = nitric oxide synthase 1, PD = Parkinson's disease.

In this retrospective analysis, 5 studies^[1,3,23,44,45]^ involving 1246 cases and 1606 controls were included to explore the link between NOS1 exon29 polymorphism and PD. As shown in Fig. 2, the combined data indicated that a lack of association was found against statistical significance between exon29 polymorphism and the susceptibility to PD under all 5 genetic models (allelic T vs C: OR = 1.11, 95%CI 0.89–1.38, *P* = 0.36; heterozygous TC vs CC: OR = 1.09, 95%CI 0.92–1.28, *P* = 0.33; homozygous TT vs CC: OR = 1.29, 95%CI 0.95–1.75, *P* = 0.10; recessive TT vs TC + CC: OR = 1.23, 95%CI 0.92–1.66, *P* = 0.16; dominant TT + TC vs CC: OR = 1.11, 95%CI 0.86–1.45, *P* = 0.42). This finding was robust enough to survive the FDR and Bonferroni–Holm correction (adjusted *P* values not shown here). Therefore, our study indicated that NOS1 exon29 polymorphism was not related to the risk of Parkinson's disease.

### 3.4 The relationship of ABCB1 1236C/T polymorphism with the predisposition to Parkinson's disease

In this study, 5 eligible studies^[18,26–29]^ containing 1124 cases and 1142 controls were collected to evaluate the relationship between ABCB1 1236C/T polymorphism and PD risk. Although no significant correlation was observed in 4 genetic models (allelic T vs C: OR = 0.93, 95%CI 0.83–1.05, *P* = 0.27; heterozygous TC vs CC: OR = 1.12, 95%CI 0.92–1.37, *P* = 0.26; homozygous TT vs CC: OR = 0.84, 95%CI 0.59–1.20, *P* = 0.34; dominant TT + TC vs CC: OR = 1.04, 95%CI 0.86–1.26, *P* = 0.68) (Table 3), ABCB1 1236C/T polymorphism was found to be statistically significantly linked with increasing or decreasing PD risk under the recessive or over-dominant model respectively (recessive TT vs TC+CC: OR = 0.80, 95%CI 0.66–0.97, *P* = 0.03; over-dominant TC vs TT+CC: OR = 1.21, 95%CI 1.03–1.43, *P* = 0.02) (Table 3). However, no statistical significance was detected in all 6 genetic models after the *P* values were adjusted following the FDR or Bonferroni-Holm correction for multiple testing (as also shown in Table 3).

### 3.5 No association between ABCB1 3435C/T polymorphism and the risk of Parkinson's disease

We collected 12 studies^[18,24–33]^ with a total of 2365 cases and 2977 controls to estimate the association between ABCB1 3435C/T polymorphism and the risk of Parkinson's disease in our meta-analysis. The combined data showed that ABCB1 3435C/T polymorphism was not associated with the susceptibility to PD in 5 genetic models (allelic T vs C: OR = 1.01, 95%CI 0.94–1.10, *P* = 0.73; heterozygous TC vs CC: OR = 0.95, 95%CI 0.83–1.09, *P* = 0.45; homozygous TT vs CC: OR = 1.04, 95%CI 0.89–1.22, *P* = 0.64; recessive TT vs TC + CC: OR = 1.07, 95%CI 0.94–1.22, *P* = 0.31; and dominant TT+TC vs CC: OR = 0.97, 95%CI 0.86–1.10, *P* = 0.91) (Table 3). No association was also observed in the Caucasians and Asians from ethnicity-based subgroup analysis (data not shown here). In addition, after applying the FDR and Bonferroni–Holm correction for multiple comparisons, the negative results from both a regular meta-analysis in the general population and each ethnicity subgroup analysis were stable and reliable (Table 3). Therefore, this study denoted that ABCB1 3435C/T polymorphism might not play a role in Parkinson's disease.

### 3.6 Tests of heterogeneity, sensitivity analyses, and publication bias

Our meta-analysis showed the existence of unobserved, moderate, or significant heterogeneity among the included studies investigating these polymorphisms. No between-study heterogeneity was found in all 5 genetic models (*I*^*2*^ = 0%) on NOS1 exon18 polymorphism (Table 3), and thus the fixed-effect model was applied to calculate their combined OR. On exon29 polymorphism (Fig. 2), moderate heterogeneity was detected in 3 genetic models (TT vs TC + CC: *I*^*2*^ = 12.40%; TT vs CC: *I*^*2*^ = 44.80%; TC vs CC: *I*^*2*^ = 45.70%) and the fixed-effect model was also used, whereas substantial heterogeneity among individual studies was discovered in 2 other genetic models (T vs C: *I*^*2*^ = 66%; TT + TC vs CC: *I*^*2*^ = 61.40%) and the random-effect model was employed. On ABCB1 1236C/T polymorphism (Table 3), an unobserved heterogeneity was detected in the heterozygous (*I*^*2*^ = 0%), dominant (*I*^*2*^ = 14.10%), and over-dominant (*I*^*2*^ = 0) models, whereas a moderate and moderate-to-significant heterogeneity were detected in the recessive (*I*^*2*^ = 47.40%) and allelic/homozygous (*I*^*2*^ = 50%/50.50%) models. All of these genetic models were performed by the fixed-effect model except that the homozygous model was evaluated by the random-effect model. Finally, on ABCB1 3435 C/T polymorphism (Table 3), the moderate heterogeneity among included studies was detected under all 5 genetic models (T vs C: *I*^*2*^ = 46%; TT vs CC: *I*^*2*^ = 38.30%; TC vs TT: 28.90%; TT vs CC + CT: 41.00%; TT + TC vs CC: *I*^*2*^ = 36.40%) and their pooled OR were calculated by the fixed-effect model. To assess whether any individual sample exerted undue influence on the risk estimate, we performed “leave-one-out” sensitivity analysis, where the overall OR and *P*-values were recalculated when each sample was removed one time. The sensitivity test indicated that no individual study notably affected the meta-analysis result. Begg's and Egger's test (Table 3) were executed to check the publication bias and no publication bias was found in our meta-analysis.

## 4 Discussion

Parkinson's disease is the second most common neurodegenerative disorder. Rare variants in monogenic forms have been identified to be connected with the disease at the gene level.^[46]^ Besides, the investigations of the associations between several candidate genes and PD as well as the genome-wide association studies have been carried out to recognize the related risk factors.^[47,48]^ Previous studies have explored the potential influences of NOS1 exon18, exon29, and ABCB1 3435C/T, 1236C/T SNP on the susceptibility to PD.^[1,3,18,23–33,44,45]^ However, the small size and the minor statistical power of the individual case-control studies led to the lack in consistency of their results. Thus, we did this meta-analysis to study the association of these 4 polymorphisms with the risk of Parkinson's disease.

Till now, 4 studies have reported the relationship of NOS1 exon18 with Parkinson's disease. Among those studies, 4 studies^[1,3,23,44]^ indicated that NOS1 exon18 polymorphism was not connected to the risk of Parkinson's disease. Our meta-analysis demonstrated that this polymorphism contributed to the disease susceptibility. The results under the allelic, homozygous, and dominant models clearly revealed that people with the TT genotype would have a higher risk of developing PD than those with genotype CC and TC. In addition, though the confidence interval of OR was across 1 in the heterozygous model (Table 3), the TC genotype might also contribute to the risk of PD. There are 3 reasons to account for it. First, only the combined OR in the heterozygous model was lack of the statistical significance. Second, the OR under the homozygous model was lower and its CI leaned more to the left than those under the recessive model, which could be caused by the potential association between the heterozygous mutant and the disease. Third, an increased nitrite content and NOS1 activity might lead to a link between the heterozygous variant of exon 18 and the development of PD.^[49,50]^ Thus, our meta-analysis results basically changed the uncorrelated results from the previous case-control studies. This is not unreasonable because the key benefit of the meta-analysis from the aggregation of information is to increase the estimator accuracy, shorten the CIs, and thus improve the statistical power to provide a better estimation, which may depart from the assessment acquired from any included study.^[51]^ However, more studies with large sample sizes are required to verify the association of NOS1 exon18 variant, especially the heterozygous TC genotype with PD risk in the future.

Currently, 5 studies have explored the correlation between NOS1 exon29 SNP and Parkinson's disease risk. Among them, 3 studies^[1,3,44]^ indicated the association whereas the other 2 studies^[23,45]^ denoted a lack of association between this polymorphism and the risk of the disease. Our meta-analysis supported that NOS1 exon29 variant did not contribute to the increase or decrease of PD. Among 3 case-control studies to show the positive association, Gupta et al^[44]^ indeed considered no relationship between NOS1 exon29 polymorphism with the PD risk although their study found the connection based on its small sample size. On the other hand, Levecque et al^[1]^ found that this polymorphism contributed to increasing the risk of sporadic PD due to an excess of haplotypes including the T allele for NOS1 exon29 in patients. However, Hague et al^[23]^ indicated that the existence of the association between NOS1 exon29 polymorphism and PD risk in the study from Levecque et al^[1]^ might be a false positive result, because the association between NOS1 exon29 and PD could not represent linkage disequilibrium with an unidentified pathogenic variant due to the constructed haplotypes across NOS1. Paul et al^[3]^ found the linkage for PD with NOS1 exon29 under the exposure of patients to commonly used OP pesticides. In fact, without considering this condition, their study indeed showed no association of exon29 minor allele with the disease (as shown in Fig. 2). Thus, it is reliable that NOS1 exon29 polymorphism was not a risk factor for PD from our analysis. However, this polymorphism might play an important role in the disease development under the gene–environment interactions (such as shown in Paul's study). In addition, the involvement of these environmental factors might cause the obvious between-study heterogeneity on this polymorphism. Hence, there is a need for us in the future to carry out additional case-control studies with gene–environment interactions for making a more precise estimation of the relationship between NOS1 exon29 SNP and Parkinson's disease.

Up to date, 5 studies have investigated the link between ABCB1 1236C/T polymorphism and the risk of PD. Among them, 3 studies^[18,27,28]^ indicated no relationship between this polymorphism and the susceptibility to the disease based on the populations of Poland, China, and Germany. The other 2 studies^[26,29]^ found the positive association of ABCB1 1236C/T variant with PD risk. Lee et al^[26]^ denoted the association due to tight linkage disequilibrium found in Asians, whereas Westerlund et al^[29]^ implied the connection of 1236C/T polymorphism with PD because of 1236C-2677G shown as a risk haplotype in Caucasians. The results from our meta-analysis indicated that this SNP might play a role in PD development. Although the allelic, heterozygous, homozygous, and dominant models revealed no association but the recessive and over-dominant showed the statistically significant correlation between ABCB1 1236C/T minor allele and the predisposition to PD. Therefore, ABCB1 1236C/T polymorphism might be connected to PD susceptibility. In addition, the opposite trends observed from a comparison between the recessive and the over-dominant models indicated that the homozygous mutant TT might decrease the development of Parkinson's disease but the heterozygous TC genotype might increase the risk of PD (Table 3). However, the statistical significance of the association under those 2 models did not remain after the *P*-values were adjusted following the False Discovery Rate or Bonferroni–Holm correction for multiple comparisons. Hence, the PD association of this polymorphism claimed in the above might be a false positive caused by type I error in statistical hypothesis testing. Nevertheless, we prefer to speculate that it might be a real positive which could appear insignificant due to the weak gene action and also which lacked sufficient evidence to help achieve a consistent conclusion due to the limit sample sizes of included studies. Because the results of the connection between 1236C/T and Parkinson's disease were not sufficiently robust to withstand statistically significant correction, further studies will be required to verify them.

Hitherto, 12 original studies have investigated the connection of ABCB1 3435C/T with the PD risk. Among them, 11 studies^[18,24,25,27–33]^ revealed that this polymorphism was not a risk factor for Parkinson's disease. The study reported by Lee et al^[26]^ found the positive association, due to their observation that haplotypes containing this polymorphism significantly modulated the risk of PD. In addition, a previous meta-analysis has explored the link between ABCB1 3435C/T and Parkinson's disease.^[52]^ Though this study found a significant association between the polymorphism and the disease, only 2 studies^[30,31]^ were included, which could lack the statistical power to find the true relationship. Thus, we performed this meta-analysis including 12 studies to reevaluate this association more accurately. Our analysis results indicated indeed a lack of association between ABCB1 3435C/T and the development of PD, which was further supported by subgroup analysis in Caucasians and Asians. This result was reliable considering that 11 out of 12 independent studies found no linkage of ABCB1 3435C/T with the risk of Parkinson's disease. Besides, the sensitivity analysis result, the moderate between-study heterogeneity and lack of publication bias also suggested that this conclusion was credible.

There were 5 advantages in this meta-analysis. First, our study is the first meta-analysis to investigate the association of NOS1 exon18, NOS1 exon29, and ABCB1 1236C/T polymorphisms with the development of Parkinson's disease. Second, though a previous meta-analysis has explored the role of ABCB1 3435C/T variant on the susceptibility to PD, our study based on a much larger sample size provided a different result from the previous work. Third, we carried out 6 genetic models including the least-frequently-used over-dominant model to assess the relationship of these SNPs with PD risk. Fourth, the results from both Begg's and Egger's tests demonstrated low risk of publication bias in this retrospective analysis. Finally, the NOS analysis indicated that all the included studies in this meta-analysis were of high quality.

Some disadvantages should not be ignored in our retrospective analysis. First, we limited the included studies to just Chinese and English literature, which might prejudice the meta-analysis results. Second, the relatively small sample size of each study might cause a restricted statistical power to identify a true relationship between the 4 polymorphisms and PD risk in this meta-analysis. Third, our analyses to only consider the suspected gene polymorphisms were based on unadjusted OR values without considering the role of other covariates such as age, gender, and exposures, which might cause our failure to detect the real association.

## 5 Conclusion

NOS1 exon18 polymorphism was a risk factor for Parkinson's disease, whereas NOS1 exon29 and ABCB1 3435C/T variants might not be associated with PD susceptibility. In addition, ABCB1 1236C/T polymorphism might be connected to Parkinson's disease but their relationship showed a little more complex. Specifically, the 2 minor alleles of the gene locus might play opposite roles in the susceptibility to PD, in which the homozygous mutant TT might decrease the development of PD but the heterozygous TC genotype might increase the risk of Parkinson's disease. However, the possible connection between 1236C/T polymorphism and PD risk did not remain statistically significant after the *P*-value was adjusted, so that more studies need to be further performed for verifying this association in the future.
